# Decision trees for early prediction of inadequate immune response to coronavirus infections: a pilot study on COVID-19

**DOI:** 10.3389/fmed.2023.1230733

**Published:** 2023-08-02

**Authors:** Fabio Pisano, Barbara Cannas, Alessandra Fanni, Manuela Pasella, Beatrice Canetto, Sabrina Rita Giglio, Stefano Mocci, Luchino Chessa, Andrea Perra, Roberto Littera

**Affiliations:** ^1^Department of Electrical and Electronic Engineering, University of Cagliari, Cagliari, Italy; ^2^BithiaTec Technologies, Elmas, Italy; ^3^Medical Genetics, Department of Medical Sciences and Public Health, University of Cagliari, Cagliari, Italy; ^4^AART-ODV (Association for the Advancement of Research on Transplantation), Cagliari, Italy; ^5^Medical Genetics, R. Binaghi Hospital, Local Public Health and Social Care Unit (ASSL) of Cagliari, Cagliari, Italy; ^6^Centre for Research University Services (CeSAR, Centro Servizi di Ateneo per la Ricerca), University of Cagliari, Cagliari, Monserrato, Italy; ^7^Department of Medical Sciences and Public Health, University of Cagliari, Cagliari, Italy; ^8^Liver Unit, Department of Internal Medicine, University Hospital of Cagliari, Cagliari, Italy; ^9^Unit of Oncology and Molecular Pathology, Department of Biomedical Sciences, University of Cagliari, Cagliari, Italy

**Keywords:** artificial intelligence, COVID-19, decision trees, disease severity, immunogenetic background, SARS-CoV-2

## Abstract

**Introduction:**

Few artificial intelligence models exist to predict severe forms of COVID-19. Most rely on post-infection laboratory data, hindering early treatment for high-risk individuals.

**Methods:**

This study developed a machine learning model to predict inherent risk of severe symptoms after contracting SARS-CoV-2. Using a Decision Tree trained on 153 Alpha variant patients, demographic, clinical and immunogenetic markers were considered. Model performance was assessed on Alpha and Delta variant datasets. Key risk factors included age, gender, absence of KIR2DS2 gene (alone or with HLA-C C1 group alleles), presence of 14-bp polymorphism in HLA-G gene, presence of KIR2DS5 gene, and presence of KIR telomeric region A/A.

**Results:**

The model achieved 83.01% accuracy for Alpha variant and 78.57% for Delta variant, with True Positive Rates of 80.82 and 77.78%, and True Negative Rates of 85.00% and 79.17%, respectively. The model showed high sensitivity in identifying individuals at risk.

**Discussion:**

The present study demonstrates the potential of AI algorithms, combined with demographic, epidemiologic, and immunogenetic data, in identifying individuals at high risk of severe COVID-19 and facilitating early treatment. Further studies are required for routine clinical integration.

## 1. Introduction

Researchers worldwide have had to face a myriad of challenges at a near-relentless pace, driven by the complex nature of the disease caused by the Severe Acute Respiratory Syndrome Coronavirus 2 (SARS-CoV-2). Although mass vaccination campaigns have significantly alleviated the effects of the pandemic, it is becoming increasingly clear that Coronavirus Disease 2019 (COVID-19) is likely to accompany us for many years to come. The vaccines have proven to be particularly effective in reducing the risk of severe and devastating forms of the disease caused by the original strain of the coronavirus. However, the emergence of new and more infectious variants of the coronavirus casts further doubts on our ability to completely eradicate the disease (1). Since the initial variant, known as Alpha (B.1.1.7), several other variants have been identified, including the Delta (B.1.617.2) and the Omicron (B.1.1.529) variants. The Delta variant has been estimated to spread about 225% faster than the Alpha variant (2). Similarly, the Omicron variant has shown increased transmissibility compared to its predecessors. As recently reported by WHO (World Health Organization), the Omicron XBB.1.16 variant has shown an increase in the prevalence, and it may become dominant in some countries and cause a rise in case incidence due to its growth advantage and immune escape characteristics. However, despite its high transmissibility, early data suggested these Omicron variants are associated with milder symptoms and a lower risk of hospitalization compared to other variants. Given that, and also the important fact that vaccines provided a significant level of protection against severe disease and other complications, the proposed study focused only on Alpha and Delta variants.

Unfortunately, up to 50% of people of all age groups end up having Post-Acute Sequelae of COVID-19 (PASC). These symptoms may linger for months or even years and seem to depend on a variety of factors including vaccination status, the virus variant and geographic region (3). It is strongly suspected that these phenomena are closely linked to the innate and adaptive immune response mechanisms of individuals (4–6).

The immune system activates different pathways of innate and adaptive immune response mechanisms to effectively combat viral infections and their associated disease progression. In previous epidemics such as SARS and MERS (Middle East Respiratory Syndrome), the immune response mechanisms of the host, including both innate and adaptive components, have been closely associated with the progression of the disease and clinical outcomes in affected patients (7). Also, in Zan et al. (8) Killer-cell Lectin-like Receptor D1 (*KLRD1*) has been identified as a potential biomarker for flu susceptibility. During the current ongoing pandemic, additional factors such as advanced age and male gender have been identified as risk factors for the progression of COVID-19 toward severe and often fatal disease (9).

The outbreak of COVID-19 has undoubtedly prompted a large number of scientists to employ Artificial Intelligence (AI) to help combat the pandemic crisis. In fact, Machine Learning and Deep Learning, particular approaches of AI, are a very useful tool to understand and fight this disease.

In literature there are several thousands of publications that show a various range of ML applications that have been developed to address different issues related to the virus and to the associated disease, including forecasting the number of future infections and the trajectory of the outbreak (10, 11); diagnosing the disease, e.g., using imaging tools in addition to the standard Polymerase Chain Reaction (PCR) test (12); predicting the mortality and severity risk (13); improving drug development and vaccination (14); enhancing contact tracing methods from individual interviews to more efficient digital contact tracing (15). What becomes apparent is that, despite the remarkable progress in the field of ML applied to COVID-19, the current approaches suffer from some important drawbacks (16–21). First and foremost, the lack of data is a crucial element in the development of models. In fact, it is well-known that patients’ data are difficult to gather, and considering that ML models rely on large datasets, this problem plays an important role on models’ generalization and performance. Secondly, many laboratory parameters included in the studies are collected after the patient has contracted the virus and the disease has already evolved, thereby excluding the option of early and tailored treatment in high-risk individuals (22, 23). Another drawback is that the proposed algorithms have not yet reached the level of a human expert. This particular flaw is closely related to the need for ML models with high interpretability and transparent output. These qualities are crucial so that human readers, e.g., medical professionals, can have trust in the conclusion made by the model. Decision Tree-based models are particularly helpful in this case as they can provide comprehensible rules behind every decision taken.

What emerges from the published studies is that ML is a popular paradigm to create new solutions that can help in the fight against the virus and the related disease. However, as far as the authors’ knowledge, no studies that can identify “*a priori*” subjects at risk of developing the severe clinical manifestation of COVID-19, based on immunogenetics, demographic and clinical characteristics, were published.

The aim of the proposed study was to develop an algorithm capable of predicting the risk of developing a more aggressive and severe disease course in individuals before they become infected with SARS-CoV-2. An AI method, combining common demographic risk factors (age and gender) (24, 25) with key immunogenetic risk markers, including *HLA* class I molecule alleles (*HLA-A, -B, -C*) and the 14-base pair polymorphism in the *HLA-G* gene, is described. In fact, classical and non-classical HLA antigens play a crucial role in activating and regulating innate and adaptive immune responses (26). Additionally, *KIR* genes regulating NK lymphocyte activity, which have a significant impact on the severity of SARS CoV-2 infection (27), have been included.

The proposed predictive model involves the use of Decision Trees (DT) (28, 29), which belong to the supervised machine learning classification methods. DTs are able to provide high classification accuracies together with a simple and intuitive representation of gathered knowledge. This peculiarity made them quite popular in different areas of medical decision making (30). DTs are compared with Naïve Bayes (NB) classifiers, as they are a common benchmark in classification problems.

Considering the above, the main contributing parts of this work are described as follows:

- Development of an algorithm: the paper presents the development of an algorithm that utilizes demographic, epidemiologic and immunogenetic data to identify individuals at high risk of developing severe clinical manifestations of COVID-19.
- Validation of the algorithm: the algorithm is validated using a limited number of cases by means of a leave-one-out cross-validation procedure, demonstrating its high predictive ability.
- Improvement in early treatment: the algorithm’s ability to identify high-risk individuals enables early treatment with antiviral drugs and passive immunotherapy, potentially improving patient outcomes and reducing the incidence of severe illness and hospitalization.
- Relevance to different COVID-19 variants: the algorithm’s effectiveness in discriminating against severe forms of COVID-19 infection is highlighted, suggesting its potential applicability and customization to new emerging variants and viruses with similar characteristics.
- Comparative analysis: the paper compares the performance of the proposed algorithm with existing approaches and discusses its advantages, highlighting the incorporation of immunogenetic markers as a distinguishing factor.
- Integration of AI in healthcare: the paper discusses the broader implications of AI in healthcare, emphasizing the integration of AI algorithms with IoMT and digital healthcare platforms. It highlights the potential of such technologies to improve disease management, diagnostics, and therapeutic strategies.

The remaining sections of the paper are structured as follows: section 2 provides a description of the Materials and Methods. This section is further divided into subsections. Section 2.1 discusses the ethical statement, section 2.2 describes the database containing the parameters of the COVID-19 infected subjects, sections 2.3 and 2.4 present a theoretical explanation of the NB and DT models, respectively, and section 2.5 introduces the indices used to assess the model’s performance. The experimental results are presented in section 3. In section 4, an organized discussion and possible limitations of the method are presented. Finally, the paper ends with a conclusion in section 5.

## 2. Materials and methods

### 2.1. Ethical statement

Patients were recruited and enrolled in the study protocol at the Department of Medical Sciences and Public Health of the University of Cagliari, the University Hospital of Cagliari (AOUCA) and the SS. Trinità Hospital of the Sardinian Regional Company for the Protection of Health (ATS Sardegna). Written informed consent was obtained from all patients and controls in accordance with the ethical standards (institutional and national) of the local human research committee. The study protocol, including informed consent procedures, conforms to the ethical guidelines of the Declaration of Helsinki and was approved by the responsible ethics committee (Ethics Committee of the Cagliari University Hospital; date of approval: 27 May 2020; protocol number GT/2020/10894). Records of written informed consent are kept on file and are included in the clinical record of each patient.

### 2.2. Clinical and laboratory parameters of the COVID-19 infected subjects

A panel of 153 patients were recruited from 1 June to 1 December 2020. The diagnosis of SARS-CoV-2 infection was confirmed in all patients by RT-PCR from nasopharyngeal swab. The patients were assigned to one of two groups according to disease severity: 73 (47.71%) patients with severe disease were assigned to the “*severe*” class and 80 (52.29%) patients, who were either asymptomatic or pauci-symptomatic, were assigned to the “*asy/pauci*” class. Patients in the *asy/pauci* group had been confined to home isolation whereas patients in the *severe* group had been hospitalized in the COVID Unit of the SS. Trinità Hospital in Cagliari. Fourteen of the 73 hospitalized patients died from cardio-respiratory arrest related to severe pulmonary impairment (interstitial pneumonia) (31).

Model aging has been observed in a variety of application scenarios. In order to test for aging of the prediction model, an additional test set was prepared using 42 patients infected by the more recent Delta variant of the coronavirus during the period from May to July 2021. Also, in this data set the diagnosis of SARS-CoV-2 infection was confirmed in all patients by RT-PCR from nasopharyngeal swab. The analysis included clinical and demographic parameters as well as the immunogenetic factors required by the model.

Most patients in the *severe* group required high-flow nasal oxygen supplementation or invasive treatment with mechanical ventilation. The second group of *asy/pauci* patients presented symptoms such as a runny nose, loss of taste or smell, headaches, a dry cough and/or other flu-like symptoms.

The clinical and demographic parameters included in the analysis were: male or female gender, age, a positive anamnesis for autoimmune diseases (Hashimoto’s thyroiditis, rheumatoid arthritis, diabetes mellitus, autoimmune hepatitis), flu vaccination performed in 2019/2020, use of levothyroxine (for thyroid disease), G6PDH deficiency, carrier of thalassemia.

Alongside these 7 clinical and demographic parameters, several immunogenetic variables based primarily on *HLA* class I and II alleles, the *14-bp insertion/deletion* polymorphism of the *HLA-G* gene, *KIR* genes and combinations of *KIR* genes and their HLA ligands were included.

More precisely, the main immunogenetic factors used for risk analysis were frequencies of the *HLA-A, HLA-B, HLA-C, HLA-DRB1* alleles, 3 *HLA-G* genotypes based on the presence of the 14-bp insertion (Ins) or deletion (Del) polymorphism of the *HLA-G* gene (*Ins/Ins, Ins/Del* or *Del/Del*) and 14 *KIR* genes (*2DL1, 2DL2, 2DL3, 2DL4, 2DL5, 3DL1, 3DL2, 3DL3, 2DS1, 2DS2, 2DS3, 2DS4, 2DS5*, and *3DS1*). Additionally, the *KIR AA* genotype (present or absent) and the most relevant functional units (combinations of *KIR* genes and HLA Class I alleles assigned to the C1, C2 or Bw4 categories of KIR ligands), i.e., *KIR2DL1/S1-HLA C2* group, *KIR2DL2/S2-HLA C1* group, *KIR3DL1/S1-HLA-Bw4* group, were included. The diversity of *KIR* haplotypes according to the four centromeric (*cA01*, *cB01*, *cB02*, *cB03*) and two telomeric (*tA01*, *tB01*) gene-content motifs (32) were also considered. To simplify the analysis, the centromeric regions were divided into *Cen A/A, Cen A/B* and *Cen B/B* and the telomeric regions into *Tel A/A Tel A/B* and *Tel B/B* (33). Finally, the role of the extended haplotype HLA-A*02:05, B*58:01, C*07:01, DRB1*03:01, which in a previous study of the Sardinian population resulted to offer protection against the severe clinical forms of COVID-19 (25), was evaluated.

Typing of the *HLA* Class I alleles (*HLA-A, HLA-B, HLA-C*), typing of *KIR* genes, and determination of the 14-bp polymorphism (*Ins/Del*) of the *HLA-G* gene were performed as described in Mocci et al., Littera et al., Amodio et al., and Caocci et al. (24–26, 34).

To construct the ML models, a database was built in which each record corresponds to one patient and each feature in these patient records corresponds to the clinical/demographic and immunogenetic parameter. Given the small number of records and the presence of missing values in the database, a record saving procedure was applied so that only features common to the 153 patients were kept. Conversely, features with no variability across the 153 patients such as *KIR2DL4, KIR3DL2*, and *KIR3DL3*, were discarded. The features kept after the cleaning procedure and their corresponding values are summarized in Table 1 (column Parameters) which also shows the presence or absence of the *HLA-A, -B, -C, -DRB1* alleles and the *KIR* genes identified in the selected sample.

**Table 1:** Clinical/demographic and immunogenetic parameters used to build the AI models and related values.

Parameters		Values
Gender		Female, Male
Age		20–96
Flu vaccination		Absent, present
Autoimmune disease		
G6PDH		
HLA-A*	01, 02, 03, 11, 23, 24, 25, 26, 29, 30, 31, 32, 33, 66, 68, 69, 80	Absent, present once, present twice
HLA-B*	07, 08, 13, 14, 15, 18, 27, 35, 37, 38, 39, 40, 41, 44, 45, 47, 49, 50, 51, 52, 53, 55, 56, 57, 58, 78	
HLA-C*	01, 02, 03, 04, 05, 06, 07, 08, 12, 14, 15, 16, 17	
HLA-DRB1*	01, 03, 04, 07, 08, 10, 11, 12, 13, 14, 15, 16	
HLA-G		*Del/Del, Ins/Del, Ins/Ins*
*KIR*	2DL1, 2DL2, 2DL3, 2DL5, 3DL1, 2DS1, 2DS2, 2DS3, 2DS4, 2DS5, 3DS1	Absent, present
Genotype AA		
HLA C1 group of epitopes		
HLA C2 group of epitopes		
KIR2DS2 and HLA C1 group		
KIR2DS1 and HLA C2 group		
Centromere (CEN)		A/A, A/B, B/B
Telomere (TEL)		
Protective Haplotype (HLA-A*02:05, B*58:01, C*07:01, DRB1*03:01)		Absent, present

The right column reports the coding adopted to construct the database for the machine learning algorithm. The possible values assigned to the first- and second-class *HLA* alleles were: absent if the allele was absent at the locus of interest; present once if only one copy of the allele was present; present twice if two copies of the allele were present.

### 2.3. Naïve Bayes

Naïve Bayes (NB) is a simple probabilistic classification technique commonly used in ML. It is based on Bayes’ theorem (29) and assumes that all features are independent of each other. In order to make prediction, the NB algorithm calculates the conditional posterior probability for each class and selects the class label with the highest probability.

In a binary classification problem, the algorithm can be further optimized by setting a proper threshold on the conditional posterior probability. If the resulting probability is above this threshold, the test instance is assigned to the positive class, otherwise to the negative class. Normally, the threshold is obtained by a cross-validation procedure in correspondence to the optimal operating point of the Receiving Operating Curve (ROC) curve (35). In this case, leave-one-out cross validation is applied. In leave-one-out cross-validation, one single observation is used to validate the remaining observations. In other words, the training process is applied once for each observation, using all the other observations as a training set and using the selected observation as the validation set (29).

### 2.4. Decision trees

DTs pertain to the class of supervised Machine Learning classification methods and were used in the proposed study to evaluate the risk for an individual to develop a severe clinical form of COVID-19. In particular, the prediction models were based on implementation adopted in the MATLAB toolbox (MATLAB R2019b) for Statistics and Machine Learning (36, 37). These Machine Learning algorithms are among the most versatile statistical models, able to automatically classify multidimensional data into two or multiple classes. In the proposed study, it was opted for a binary classification for the prediction of asymptomatic/pauci-symptomatic (*asy/pauci* class = 0) or severe (*severe* class = 1) disease.

DTs consist of hierarchical data structures that grow through a divide-and-conquer strategy (28) categorizing data samples into different classes. The construction of a DT starts from the root node, which contains the entire dataset with a class composition reflecting the proportion among class populations in the whole dataset. In the proposed problem, 48% of the instances belong to the *severe* class, whereas the remaining 52% belong to the *asy/pauci* class. At each node, the algorithm selects the best feature to split the data based on a certain criterion, such as minimizing the impurity of that node. Two of the most popular impurity indexes are Entropy and Gini’s Diversity Index, calculated, respectively, as follows:

(1) H(N)=−Pr(1|N)log2Pr(1|N)−Pr(O|N)log2Pr(O|N)

(2) G(N)=2Pr(1|N)Pr(O|N)

where Pr(1|*N*) and Pr(0|*N*) represent the conditional probabilities of an instance belonging to *severe* and *asy/pauci* classes. These measures are used to evaluate the homogeneity of the labels at the node and guide the DT algorithm in determining the best split to create more homogeneous child nodes.

**Figure 1:**
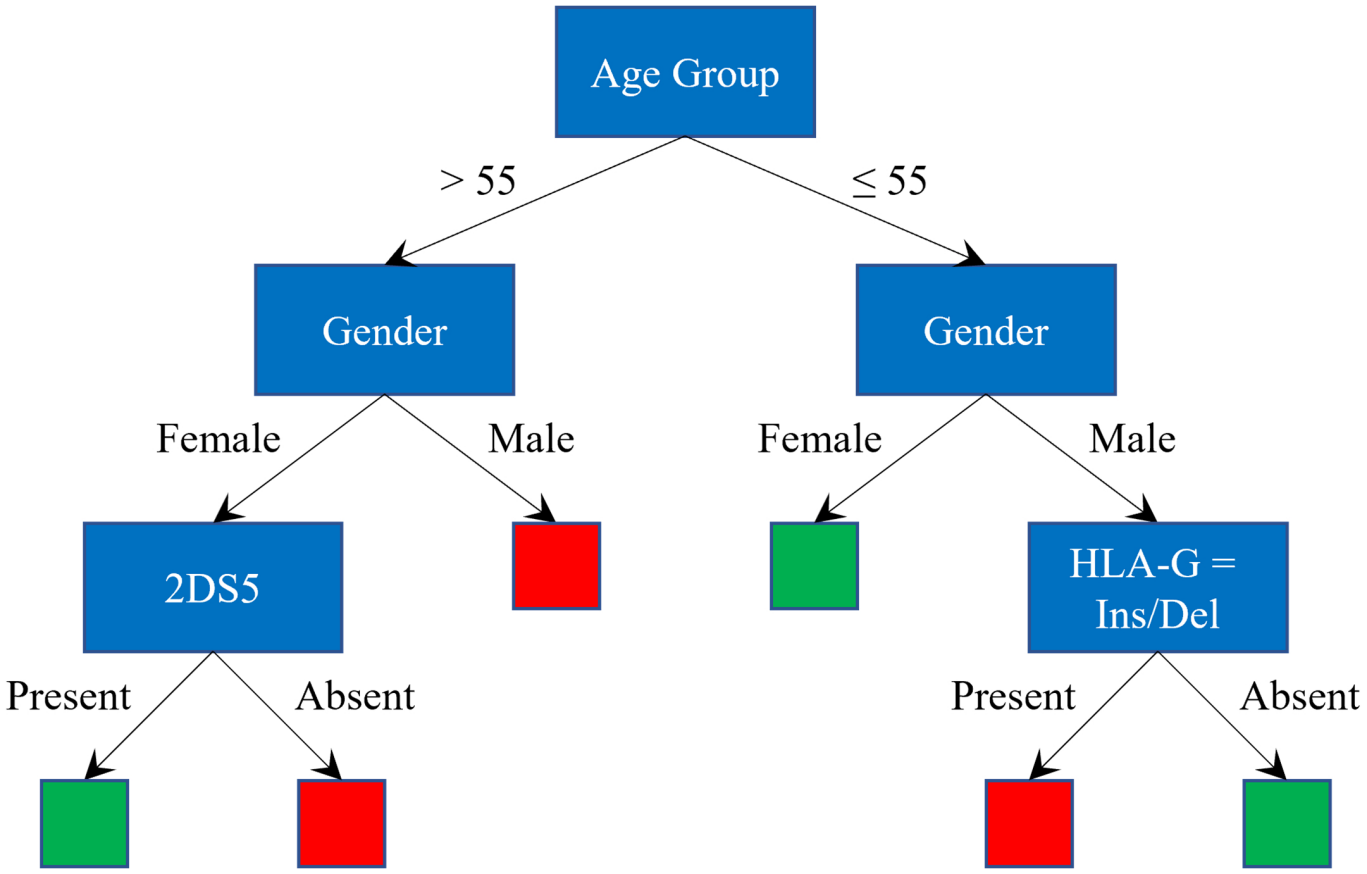
Graphical representation of a Decision Tree. Red and green leaves indicate a classification as *severe* and *asy/pauci*, respectively.

The splitting process continues recursively until leaf nodes are reached, where final decisions are taken, as shown in Figure 1, where red and green leaves indicate a classification as *severe* and *asy/pauci*, respectively.

Once the decision tree is constructed, a decision on a test sample can be reached by simply following the conditions in the tree until a leaf is reached. Based on the training set distribution, a posterior probability is assigned to the leaf and a score is assigned to the test sample. If the score is above (or below) a certain threshold, the test instance is assigned to the positive (or negative) class. Also in this case, the optimum value of the threshold is normally obtained by leave-one-out cross-validation procedure.

There are several motivations that support the choice of DTs among the numerous and well-known ML classification methods, such as Neural Networks (NNs), SVM, discriminant analysis, and so on (29). The main reasons backing the decision to apply DTs were:

- features can be both continuous or categorical (in the problem at hand the features are categorial with binary or multiple values);
- availability of large training datasets is not mandatory, as opposed to other machine learning methods (as shown in paragraph 2.2, in the proposed problem, a limited number of instances are available);
- the contributions of the features can be easily evaluated; and
- when using the model on an instance never presented before, the final decision (i.e., the classification of an instance) is easy to understand, interpret and visualize, simply by following the rules used along the path from the root to the leaf.

### 2.5. Methodology

Firstly, univariate analysis was performed on all clinical/demographic and immunogenetic parameters, to find the parameters more related to both low and high risk of developing a severe form of COVID-19. The age, which maximized the separation between *asy/pauci* and *severe* groups, was used as a threshold to calculate the odds ratios (OR).

Secondly, the training of the DT classifiers was performed. Given the limited size of the database, 10 training sessions were performed selecting 10 training and 10 test set sets by means of the Bootstrap Aggregating (Bagging) procedure. Bagging reduces the eventual bias arising from the choice of a particular test set (38). By subsampling the initial database of 153 patients, 10 balanced test sets of 30 patients were created, each one composed of 15 *asy/pauci* patients and 15 *severe* patients, none of whom were used for the model training process. For each test set, a model was trained on the remaining 123 patients, 65 pertaining to the *asy/pauci* group and 58 to the *severe* group. Subsequently, predictive performance was evaluated by averaging the performance of the 10 models. For the validation of the models, leave-one-out cross-validation was used.

One of the advantages of using DTs is the possibility to derive rules easily interpretable by the medical staff. In order to create a single model capable of improving the classification based exclusively on age group and gender, from each of the 10 DT models, the rules leading to classification scores greater than 2/3 on the training set were extrapolated. Only the rules present in at least three models were retained.

Aging of the prediction model was evaluated by preparing an additional test set of 42 patients infected by the highly contagious Delta variant of SARS-CoV-2. The clinical and demographic parameters were included in the analysis as well as the immunogenetic factors required by the model.

### 2.6. Performance indexes

In the following section, performance of the classifiers was tested on the database described in paragraphs 2.1 and 2.2. The performance indexes used to evaluate the classifier models were the well-known metrics as: True Positive Rate (TPR) or sensitivity, which refers to the ability of the model to correctly detect *severe* patients; True Negative Rate (TNR) or specificity, which refers to the ability of the model to correctly detect asy/pauci patients; Accuracy (ACC), which refers to the proportion of correctly classified patients among all cases; Positive Predictive Value (PPV) and Negative Predictive Value (NPV), which measure the proportion of true positives (TP) among the positive test results and the proportion of true negatives (TN) among the negative test results, respectively.

## 3. Results

**Figure 2:**
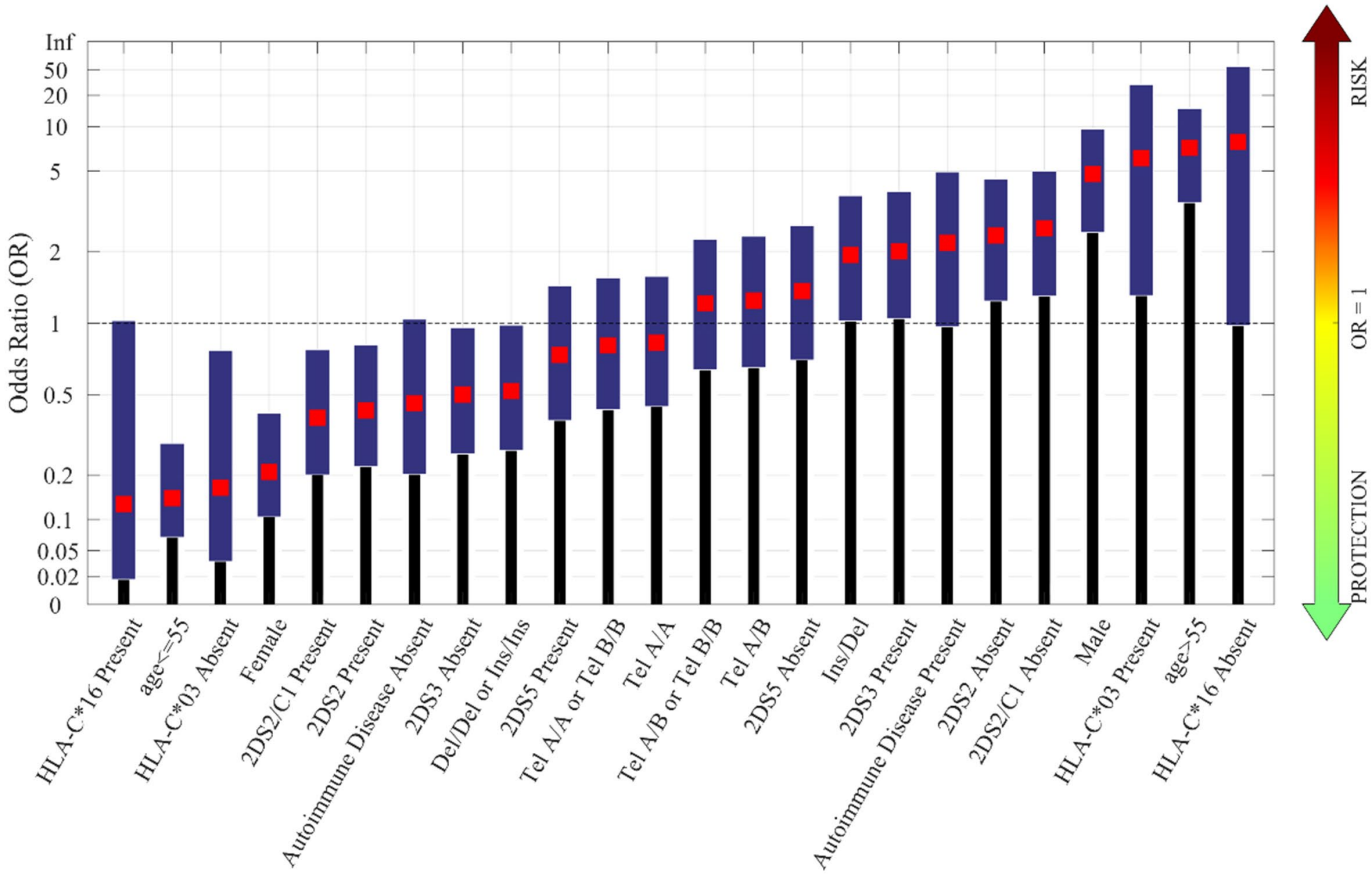
Odds-ratio of factors that influence the severity of COVID-19 disease in Sardinian patients (odds ratio: red squares, blue bars: 95% confidence interval).

Figure 2 shows the odds ratio for COVID 19 protective and risk factors and their related confidence intervals (39). The plots highlight the factors more closely associated with protection or an increased risk of a severe clinical disease course. The age, which maximized the separation between *asy/pauci* and *severe* groups, was 55.

The immunogenetic factors that seemed to offer increased protection against the more severe clinical forms of COVID 19 were: age less than or equal to 55 years, female gender, presence of the KIR2DS2 gene, particularly when combined with HLA-C alleles of the C1 group, presence of KIR2DS3 and presence of the HLA-G 14-bp Del/Del or Ins/Ins genotype. It is interesting to note that the presence of the protective haplotype (HLA-A*02:05, B*58:01, C*07:01, DRB1*03:01), not shown in Figure 2, yielded an OR of 0, owing to the fact that this haplotype was completely absent in the *severe* group of patients. This factor has previously been shown to have a strong protective effect and, despite its relatively high frequency (2.6%) in the Sardinian population, was only observed in 4 of the 80 (3%) individuals pertaining to the asy/pauci group.

The strongest risk factors for a severe clinical disease course were: age above 55 years, male gender, absence of the KIR2DS2 gene alone or in combination with HLA-C alleles of the C1 group (KIR2DS2/HLA-C C1+), presence of the 14-bp polymorphism (Ins/Del) of the HLA-G gene, presence of the KIR2DS5 gene and presence of the KIR telomeric region A/A (Tel A/A).

The associations found for COVID 19 protection/risk factors using univariate analysis deserve further investigation in larger cohorts of patients.

Table 2A shows the average performance of the 10 NB models for training, leave-one-out, and test data sets. The range of the different indices among the 10 models is also provided for the whole database. The NB models, trained on the full set of features, showed relatively high accuracy on the training data with a score of 86.02%; despite this, the average accuracy for leave-one-out and test were 61.87% and 60.33%, respectively. This shows that NB models fit the training data, but perform worse on test data, which means that they experience a high overfitting. Similar results would be obtained by means of DT without any action to limit overfitting.

**Table 2:** Average performance of (A) NB models, (B) DT models, (C) NB models with selected features from DTs, for training, leave-one-out and test sets.

	Training	Leave-One-Out	Test	Whole
**(A) NB models**				
P	58	58	15	73
TP	49.0	32.0	8.6	57.6 (47–63)
TPR (%)	84.48	55.17	57.33	78.90 (64.38–86.30)
N	65	65	15	80
TN	56.8	44.1	9.5	66.3 (61–70)
TNR (%)	87.38	67.85	63.33	82.87 (76.25–87.50)
P + N	123	123	30	153
TP + TN	105.8	76.1	18.1	123.9 (115–131)
ACC (%)	86.02	61.87	60.33	80.98 (75.16–85.62)
PPV (%)	85.66	60.49	61.00	80.96 (75.00–85.92)
NPV (%)	86.32	62.91	59.80	81.33 (72.34–86.30)
**(B) DT models**				
P	58	58	15	73
TP	48.1	47.0	10.9	59.0 (53–62)
TPR (%)	82.93	81.03	72.67	80.82 (72.60–84.93)
N	65	65	15	80
TN	54.5	52.6	11.5	66.0 (60–71)
TNR (%)	83.85	80.92	76.67	82.50 (75.00–88.75)
P + N	123	123	30	153
TP + TN	102.6	99.6	22.4	125.0 (122–127)
ACC (%)	83.41	80.98	74.67	81.70 (79.74–83.01)
PPV (%)	82.45	79.30	76.31	81.07 (75.61–85.94)
NPV (%)	84.83	83.00	73.86	82.66 (78.02–85.14)
**(C) NB models with selected features from DTs**				
P	58	58	15	73
TP	48.7	44.6	11.4	60.1 (56–66)
TPR (%)	83.97	76.90	76.00	82.33 (76.71–90.41)
N	65	65	15	80
TN	54.3	50.1	11.4	65.7 (61–71)
TNR (%)	83.54	77.08	76.00	82.12 (76.25–88.75)
P + N	123	123	30	153
TP + TN	103.0	94.7	22.8	125.8 (121–132)
ACC (%)	83.74	76.99	76.00	82.22 (79.08–86.27)
PPV (%)	81.99	74.96	76.00	80.92 (77.21–87.71)
NPV (%)	85.38	78.90	76.00	83.71 (79.27–89.71)

The same performance and corresponding ranges (in brackets) obtained on the 10 DT models are also provided for the whole dataset.

**Figure 3:**
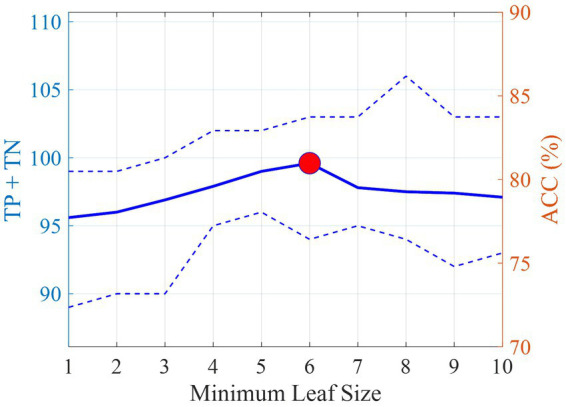
Average number of correct classifications (TP + TN) and accuracy (ACC) on the leave-one-out set with varying minimum sample size on the terminal nodes. The dotted lines indicate the minimum and maximum values obtained in the 10 DT models. The red circle indicates the best accuracy obtained and the corresponding minimum leaf size.

To avoid overfitting, when training the DT models, the tree-building process was stopped before it produced leaves with very small samples. This heuristic is known as early stopping, although occasionally it is referred to as pre-pruning. The minimum sample size in terminal nodes was fixed at 6, since this helps to maximize the average accuracy on the 10 models for the leave-one-out set (see Figure 3). An example of a pre-pruned tree (one of the 10 trained models) is shown in Figure 1.

Table 2B shows the average performance of the 10 DT models for training, leave-one-out and test data sets. The range of the different indices among the 10 models is also provided for the whole database. With respect to the results obtained on NB models, the DT models showed similar accuracy on the training data with a score of 83.41%, while higher average accuracy, equal to 80.98%, for leave-one-out. Accuracy on test data decreased on average to 74.67%showing also for decision trees a little overfitting. When considering performance on the whole dataset, the averaged accuracy remained high (81.70%). The accuracy in the worst model was still high (79.74%). However, larger deviations were observed for TPR (72.60–84.93) and TNR (75.00–88.75). In fact, models with low TPR generally report high TNR and vice versa, making accuracy values quite stable.

**Figure 4:**
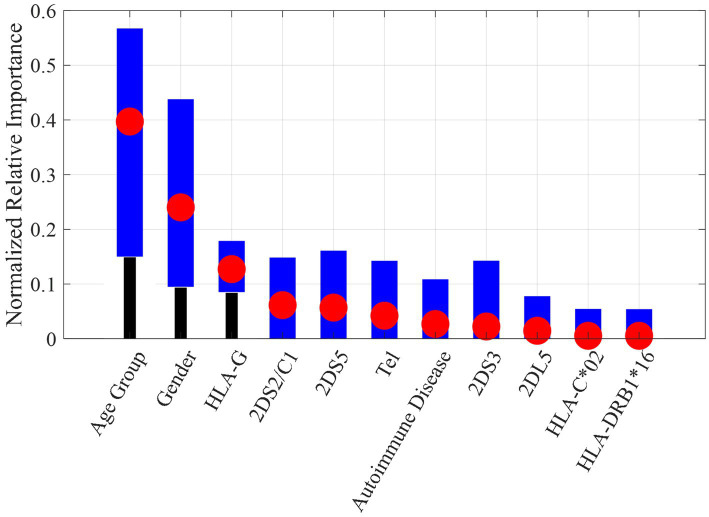
Normalized relative importance for each feature averaged among the 10 DT models (red circle) with min-max interval (blue bars).

The reduced overfitting obtained on DT models with respect to NB models could be due, as well as to the pre-pruning stage, also to the capability of DTs to perform a feature selection. Figure 4 shows the relative importance of the most significant features (in order of importance) used by the DT models. In order to easily compare the importance obtained in the different tests, the impurity gain for each feature was normalized by the sum of the impurity gains of all features. The red circle indicates the average normalized importance, while the blue bars indicate the interval obtained among the 10 models.

As can be seen, the most important features were age group and gender. This finding is furthermore supported by the results of univariate analysis shown in Figure 2.

The genetic feature with the highest importance was the 14-bp insertion/deletion polymorphism of the *HLA-G* gene, already identified as a risk or protection factor in univariate analysis. For the sake of comparison, the results on the NB models by using only the 11 features selected by DTs shown in Figure 4 are reported in Table 2C. As it can be noticed, the performances on the leave-one-out after the feature selection are better than those obtained with the full features dataset and closer to the results obtained by DT models.

In line with current literature, age group and gender were leading predictors for all the considered models. The age group threshold identified by the DTs was 55. Table 3 reports the number of *asy/pauci* and *severe* classified cases according to the different age and gender groups. In the database, the *asy/pauci* and *severe* cases were equinumerous (19 *asy/pauci* and 18 *severe*) in the group of women over 55 years of age and, therefore, did not provide any information for this group. On the other hand, 40 out of the 49 men older than 55 developed severe symptoms. Among younger patients (age less than 56 years), only 2 of the 35 women developed severe symptoms. Thus, young age is in itself a protection factor for women. Among younger men, only 13 out of 32 developed severe symptoms.

**Table 3:** Database grouping based on age and gender.

Age group	Gender	*Asy/pauci*	Severe
>55	F	19	18
	M	9	40
≤55	F	33	2
	M	19	13

After extrapolating the rules leading to classification scores greater than 2/3 on the training set, present in at least three models, the retained rules involved four genetic parameters: presence or absence of the *HLA-G 14-bp Del/Ins* genotype, presence or absence of *KIR2DS5*, presence or absence of 2DS2/HLA-C1 and presence of the KIR telomeric region (Tel A/A). Among older women, addition of the parameter *KIR2DS5* allowed for correct classification in 27 out of 37 cases: 14 out of 19 *asy/pauci* cases and 13 out of 18 *severe* cases. Among older men, after adding presence or absence of the KIR2DS2/HLA-C C1+ functional unit and the KIR telomeric region (Tel A/A), it became possible to correctly classify 42 out of 49 cases: 5 among the 9 *severe* cases and 37 among the 40 *asy/pauci* cases. In the group of younger men, presence or absence of the *HLA-G 14-bp Del/Ins* genotype allowed for the correct classification of 25 out of 32 cases: 9 among the 13 *severe* cases and 16 among the 19 *asy/pauci* cases.

**Figure 5:**
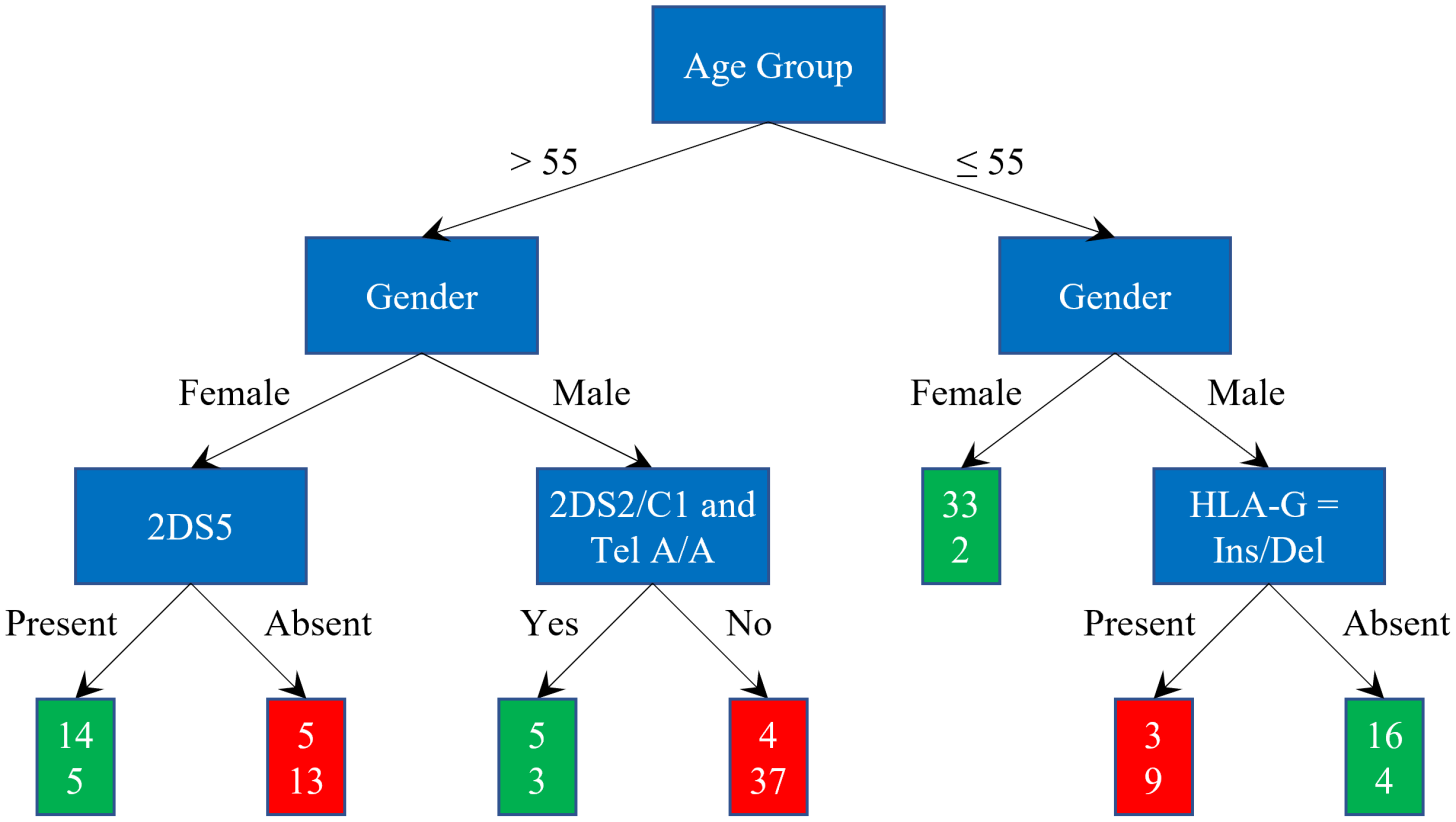
Classification scheme and results: green and red rectangles represent the *asy/pauci* and *severe* classes respectively; the two numbers are the number of *asy/pauci* and *severe* patients, respectively, falling within the class.

Figure 5 illustrates the diagnosis classification scheme. The red and green rectangles represent *severe* and *asy/pauci* classification, respectively. The two numbers in these rectangles indicate the number of *asy/pauci* and *severe* patients falling within each class. Overall, 126 out of 153 cases were correctly classified: 63 of the 80 *asy/pauci* cases and 63 of the 73 *severe* cases. The classification failed for 10 older women (5 *asy/pauci* and 5 *severe*), 7 older men (4 *asy/pauci* and 3 *severe*), 2 *severe* young women and 7 young men (3 *asy/pauci* and 4 *severe*). As shown, for older women, older men and younger men, the additional genetic parameters improved the classification based on age group and gender by about 21.62%, 4.08%, and 20.00%, respectively.

Performance of the two models—one based on age group and gender and the other expanded to include genetic parameters—is shown in Table 4A. Although the model based on age group and gender has a quite good performance in recognizing actual *asy/pauci* patients, it exhibits a very low ability in recognizing actual *severe* patients. The inclusion of genetic parameters helps to increase the accuracy on the overall dataset by more than 10 % points, thereby consistently increasing the TPR from 54.79% to 80.82%. This property is confirmed by the PPV and NPV. In fact, while the classification based on age group and gender has a low NPV, demonstrating a low ability in identifying *asy/pauci* cases, the proposed expanded model yielded both PPV and NPV above 80%.

**Table 4:** Performance on the (A) Alpha variant infected patients and (B) Delta variant infected patient datasets of the two models: one based exclusively on age group and gender and a proposed model expanded to include four relevant genetic parameters.

	(A) Alpha variant infected patients		(B) Delta variant infected patients	
	Age group and gender	Proposed model	Age group and gender	Proposed model
P	73	73	18	18
TP	40	59	8	14
TPR (%)	54.79	80.82	44.44	77.78
N	80	80	24	24
TN	71	68	22	19
TNR (%)	88.75	85.00	91.67	79.17
P + N	153	153	42	42
TP + TN	111	127	30	33
ACC (%)	72.55	83.01	71.43	78.57
PPV (%)	81.63	83.10	80.00	73.68
NPV (%)	68.27	82.93	68.75	82.61

The test on the aging of the prediction model in Figure 5 by means of the 42 patients infected by the Delta variant, lead to 24 (57.14%) patients with severe disease assigned to the *severe* class, and 18 (42.86%) patients, who were either asymptomatic or pauci-symptomatic, assigned to the *asy/pauci* class. Out of the 18 female patients, most of whom were under the age of 55, 77% did not develop severe disease. Out of the remaining 24 male patients, 58.3% developed the severe form of the disease.

Table 4B compares the performance of the proposed predictive model to that of the model exclusively based on age group and gender. Although the model based on age group and gender achieved quite good performance in identifying *asy/pauci* patients, it had a very low ability in recognizing severely ill patients. After adding the genetic parameters, accuracy increased by more than 7 % points with a remarkable increase in TPR from 44.44% to 77.78%. Moreover, TNR% yielded a value greater than 79%. It should be noted that the proposed model also yielded quite good values for PPV and NPV. The latter reached a value of about 83% thereby providing us with a high degree of confidence that, in case of negative predicted value, the tested patient will not develop the severe form of the disease even if he/she has been infected by the Delta variant virus. It is worth noting that PPV and NPV are closely related to the prevalence, and thus should be carefully evaluated during screening stages. The comparison with the numbers in Table 4A confirms the conclusions made when referring to the datasets of patients infected by the Alpha variant, thereby showing a limited aging effect; all the performance indices decreased but still remained well above the 70% threshold.

## 4. Discussion

Medicine is one of the main sectors in which the use of AI has been successfully applied, not only achieving improvement in diagnostic strategies but also enabling more specific and targeted therapies that in future will allow for increasingly “personalized” medicine. The application of AI embraces a variety of medical fields that range from the digitalization of diagnostic imaging and the replacement of paper files with digital reporting to the development of biotechnologies associated to “omics” sciences, all of which have led to a true explosion of the so-called Internet of Medical Things (IoMT). IoMT is focused on healthcare and is an extended and more specific version of the Internet of Things (IoT) (40–42). Combined to point-of-care diagnosis, it could be used within the current crisis to develop a digital healthcare platform capable of administering proper care to COVID-19 patients at home as well as to provide government and healthcare organizations with a comprehensive disease management database (43).

On the topic of prediction of mortality risk and severity, several works have been published. The aim of the first line of research was to assess the mortality risk, i.e., to determine patients with the highest risk of dying from COVID 19 using clinical, demographic and radiological data on admission to the hospital from the electronic medical records (44–48). Each study selected various biomarkers that can predict the mortality, e.g., age, male gender, white blood cell, comorbidity profile of the patient such as diabetes mellitus, respiratory distress, coronary artery disease, and others.

The second line of research aforementioned, focused on the assessment of the severity risk, i.e., the development of a ML model based on clinical, radiological and laboratory characteristic to predict, on the basis of the patients outcome, the severity of the disease (49–53). Male gender, obesity, smoking, cerebrovascular disease, chronic liver disease, etc., were clinical determinants of Covid-19 severity. The study in Toraih et al. (54) explored pre-existing cardiovascular diseases as a risk factor of a severe form of COVID 19. This study suggested that, beyond the well-known pulmonary complications, high severity and mortality rates are closely related to viral myocarditis, myocardial damage, microangiopathy, and acute coronary syndromes. The conclusion of this study was that elevated cardiac injury biomarkers may improve the identification of patients with high risk of mortality and severity. In de Freitas et al. (55)’, the main symptoms, signs and demographic data that were most often associated to the hospitalization in patients with respiratory problems, have been defined by means of a ML model. In Sun et al. (56), researchers employed a Support Vector Machine (SVM) model where input data are associated with the clinical course of COVID-19. The results showed that a four-feature combination of GSH, CD3 ratio, total protein and age resulted in an Area Under Receiving Operating Curve (AUROC) of 0.9996 and 0.9757 in the training and testing datasets, respectively. Kaplan–Meier survival and cox-multivariate regression analyses confirmed the ability of the model to individuate severe cases using the selected clinical parameters. Moreover, after evaluating 253 clinical blood samples from the city of Wuhan in China, a group of researchers found 11 key relevant indices (total bilirubin, creatine kinase isoenzyme, glucose, creatinine, kalium, lactate dehydrogenase, platelet distribution width, calcium, basophil, total protein, and magnesium) which can detect, with an overall specificity and sensitivity of 95.95 and 96.97%, respectively, COVID 19 infected patients (57).

In general, most of these algorithms couple demographic parameters such as the sex and age of the patients to simple clinical and laboratory parameters such as blood group, transaminase, bilirubin, creatinine, number of platelets, etc. Some other studies have included more sophisticated laboratory markers: the dosage of cytokines, chemokines, the structure of lymphocyte subpopulations and various genetic factors including mutations in coagulation genes (58, 59). In Yasar et al. (60), three COVID-19 positive patient groups (mild, severe, and critical) and a control group have been classified based on the blood protein profiling.

Among all ML methods, Logistic Regression, SVM, Random Forest (RF) and Gradient Boosting Decision Trees were the most used. Particularly, the best possible results may be achieved by the gradient boosting models, RF and NNs.

Additionally, machine learning models tuned by metaheuristic algorithms like Genetic Algorithms and Particle Swarm Optimization have gained popularity due to their ability to find near-optimal solutions in complex problem domains.

In the context of COVID-19, metaheuristic frameworks that combine the Generalized Boltzmann distribution and orthogonal Jacobi polynomials have been successfully employed to analyze the spread of the virus (61). These metaheuristic approaches have also found applications in other critical areas, such as the monitoring and management of COVID-19 patients. By leveraging the Internet of Things (IoT), real-time monitoring of COVID-19 patients could be achieved through wearable devices, enabling automatic alerts to mitigate risk factors (62).

Throughout the pandemic, millions of people with or without symptoms have been isolated at home, waiting for the evolution of the infection either toward recovery or worsening of the clinical symptoms. This has often led to delays in hospitalization and the administration of timely and effective treatment (63). Indeed, early treatment of patients at a greater risk of developing the more severe forms of the disease is fundamental to the healing process and reduces the incidence of patients admitted to intensive care and the overall mortality rate (64).

AI algorithms have been widely used to predict the clinical evolution of SARS-CoV-2 infection. These algorithms have a high accuracy and ability to discriminate severe cases. However, an intrinsic limitation is represented by the fact that the laboratory and instrumental parameters on which these tools are based are altered by the interaction of the organism with the virus. This limits their predictive ability in the early stages of infection, when it is more important to be able to predict the evolution of the disease toward severe forms in order to ensure timely and appropriate treatment strategies.

The antiviral Paxlovid (Pfizer Inc.) for COVID-19 appear promising for treating COVID-19 in the early stages and seem to be particularly indicated for adults with mild to moderate disease and at risk of developing a more severe disease course (65). Therefore, it becomes even more important to identify individuals who are at a high risk of experiencing severe illness and hospitalization.

Unlike previous algorithms described in the literature, the proposed algorithm is based on demographic and epidemiologic data as well as the specific immunogenetic characteristics of each individual which are totally unrelated to the humoral and tissue changes generated during the viral infection.

This approach, which has a TPR and TNR of around 80%, allows us to identify “*a priori*” subjects at high risk of developing the severe clinical manifestations of COVID-19 should they become infected with SARS-CoV-2. Despite the limited number of cases used for AI training, the extremely high predictive ability of the model supports the reliability and effectiveness of the proposed algorithm.

The high ability to discriminate subjects at high risk of developing the severe and potentially lethal forms of COVID-19, both before and in the early stages of SARS-CoV-2 infection, makes this particular algorithm an interesting system for use in clinical practice. Another problem that needs to be addressed is that even fully vaccinated individuals do not always mount an appropriate immune response when challenged with the virus and in some cases may require booster shots to ensure adequate protection. The proposed algorithm might be capable of individuating these cases and could perhaps help researchers upgrade therapies as well as the trained immunity currently offered by COVID-19 vaccines.

The main limitation of this study lies in the relatively small number of subjects examined, as well as the focus on only two variants [B.1.1.7 (Alpha) and B.1.617.1 (Delta)]. Therefore, the high predictive capabilities of the proposed AI algorithm need to be validated on larger and more diverse cohorts. Furthermore, the use of these approaches is currently hindered by the challenge of regularly collecting immunogenetic variables in non-hospitalized patients, thus limiting its application beyond hospital settings. However, it is important to acknowledge the growing trend of integrating genomic information into routine clinical practice. Technological advancements and decreasing costs of genomic sequencing are making complete genomic profiling of individuals at birth increasingly feasible and potentially commonplace. As genomic medicine progresses, the availability and integration of immunogenetic variables in clinical settings are expected to improve, creating new opportunities for the broader application of AI algorithms in healthcare.

However, the algorithm can be significantly simplified by reducing the number of immunogenetic markers to only four genetic parameters strongly associated with a more severe disease course: absence of the *KIR2DS2* gene alone or in combination with *HLA-C* alleles of the C1 group (KIR2DS2/HLA-C C1+), presence of the 14-bp polymorphism (*Ins/Del*) of the *HLA-G* gene, absence of the *KIR2DS5* gene and absence of the *KIR* telomeric region A/A (Tel A/A).

These four genetic markers made it possible to identify with certainty about 80% (TPR) of all patients who developed the severe form of COVID-19 whereas the use of sex and age alone only identified half of those at risk of developing severe SARS-CoV-2 infection.

It is interesting to note that the proposed algorithm resulted to be effective in discriminating against severe forms of COVID19 infection independent of whether the patients had been infected by the Alpha variant and the more common and widespread Delta variant. It can therefore be assumed that its application would also be effective in the case of newly emerging Omicron variants.

In synthesis, all subjects who test positive by a nasopharyngeal swab can quickly be classified in a low or high-risk range. Those at high risk can receive early treatment with the anti-COVID19 drugs such as the oral antiviral drug Molnupiravir (66) and/or passive immunotherapy based on the use of anti-spike monoclonal antibodies which has shown to yield the best results when administered in the initial stages of infection (63, 67).

The proposed algorithm could be made user-friendly by creating a rapid antigen test to analyze the four most relevant genetic markers either at home or in the clinic. The algorithm can be inserted into an application for iOS or Android to facilitate its use even in an out-of-hospital setting. This would allow for early treatment of patients at high risk of developing severe disease with the newly emerging but highly expensive drugs.

However, considering that it is extremely difficult to develop high quality clinical prediction models that are beneficial to patients and providers in the different areas of healthcare (22), the proposed algorithm will need to undergo scrupulous validation on large cohorts before it can be recommended for widespread use in routine clinical practice.

Overall, the algorithm tested in this pilot study is extremely reliable and combines clinical and demographic features with some simple immunogenetic markers associated with the more severe forms of COVID-19. With appropriate modifications, the proposed approach could represent a starting point for the creation of other algorithms applicable to a wide spectrum of viral infections.

## 5. Conclusion

In conclusion, the application of artificial intelligence (AI) in the field of medicine, particularly in the context of the COVID-19 pandemic, has shown great potential for improving diagnostic strategies and enabling personalized medicine. The use of AI algorithms has facilitated the prediction of clinical evolution and identification of individuals at high risk of developing severe forms of COVID-19. While existing AI algorithms primarily rely on laboratory and instrumental parameters that are influenced by the interaction between the virus and the organism, the proposed algorithm takes into account demographic, epidemiologic, and specific immunogenetic characteristics. This unique approach has demonstrated a high accuracy in identifying individuals at high risk of severe clinical manifestations of COVID-19, even in the early stages of infection. Furthermore, the algorithm’s ability to discriminate between severe and mild/moderate cases of COVID-19 is independent of the viral variants, including the Alpha and Delta variants, suggesting its potential effectiveness against variants and viruses with the same characteristics. To simplify the implementation of the algorithm in clinical practice, it is proposed to reduce the number of immunogenetic markers to four genetic parameters strongly associated with a more severe disease course. This would make it feasible to determine the risk level of patients using a rapid antigen test in a user-friendly manner, either at home or in a clinic setting. However, before widespread implementation, the algorithm requires rigorous validation on large cohorts to ensure its reliability and effectiveness. Additionally, the integration of the algorithm into a digital healthcare platform, along with the administration of early treatment using antiviral drugs and passive immunotherapy, could significantly improve disease management and reduce the incidence of severe cases and overall mortality rate. In conclusion, the present study demonstrates the potential of AI algorithms, combined with demographic, epidemiologic, and immunogenetic data, in identifying individuals at high risk of severe COVID-19 and facilitating early treatment. This approach could serve as a foundation for the development of similar algorithms for various viral infections, highlighting the broader applications of AI in healthcare.

## Data availability statement

The datasets presented in this study can be found in online repositories. The names of the repository/repositories and accession number(s) can be found at: https://www.ncbi.nlm.nih.gov/bioproject/PRJNA904643.

## Ethics statement

The studies involving human participants were reviewed and approved by Ethics Committee of the Cagliari University Hospital; date of approval: 27 May 2020; protocol number GT/2020/10894. The patients/participants provided their written informed consent to participate in this study.

## Author contributions

All authors listed have made a substantial, direct, and intellectual contribution to the work and approved it for publication.

## Funding

This study was partially funded by the Italian Ministry of University and Research, PNRR, mission 4, component 2, investment 1.3 (Partenariati estesi alle università, ai centri di ricerca, alle aziende per il finanziamento di progetti di ricerca di base), title HEAL ITALIA, project number PE00000019, CUP: F53C22000750006 (AP, University of Cagliari). This research was supported by “Fondazione di Sardegna,” grant no. 2023.0160 to the non-profit organization “Associazione per l’Avanzamento della Ricerca per i Trapianti (AART-ODV)”.

## Conflict of interest

BeC was employed by BithiaTec Technologies.

The remaining authors declare that the research was conducted in the absence of any commercial or financial relationships that could be construed as a potential conflict of interest.

## Publisher’s note

All claims expressed in this article are solely those of the authors and do not necessarily represent those of their affiliated organizations, or those of the publisher, the editors and the reviewers. Any product that may be evaluated in this article, or claim that may be made by its manufacturer, is not guaranteed or endorsed by the publisher.
